# Coping with COVID-19: medical students as strong and responsible stewards of their education

**DOI:** 10.1007/s40037-021-00650-3

**Published:** 2021-01-25

**Authors:** Jacquelyn B. Kercheval, Deena Khamees, Charles A. Keilin, Netana H. Markovitz, Eve D. Losman

**Affiliations:** 1 Medical School, University of Michigan, Ann Arbor, MI USA; 2 Department of Emergency Medicine, University of Michigan, Ann Arbor, MI USA

**Keywords:** Curriculum development, Pandemic, Student-driven, Adult learning theory, Master Adaptive Learner framework

## Abstract

**Background:**

Due to the COVID-19 pandemic, clinical rotations at the University of Michigan Medical School (UMMS) were suspended on March 17, 2020, per the Association of American Medical Colleges’ recommendations. No alternative curriculum existed to fill the educational void for clinical students. The traditional approach to curriculum development was not feasible during the pandemic as faculty were redeployed to clinical care, and the immediate need for continued learning necessitated a new model.

**Approach:**

One student developed an outline for an online course on pandemics based on peer-to-peer conversations regarding learners’ interests and needs, and she proposed that students author the content given the immediate need for a curriculum. Fifteen student volunteers developed content to fill knowledge gaps, and expert faculty reviewers confirmed that the student authors had successfully curated a comprehensive curriculum.

**Evaluation:**

The crowdsourced student content coalesced into a 40-hour curriculum required for all 371 clinical-level students at UMMS. This student-driven effort took just 17 days from outline to implementation, and the final product is a full course comprising five modules, multiple choice questions, discussion boards, and assignments. Learners were surveyed to gauge success, and 93% rated this content as relevant to all medical students.

**Reflection:**

The successful implementation of this model for curriculum development, grounded in the Master Adaptive Learner framework, suggests that medical students can be entrusted as stewards of their own education. As we return to a post-pandemic “normal,” this approach could be applied to the maintenance and de novo development of future curricula.

**Supplementary Information:**

The online version of this article (10.1007/s40037-021-00650-3) contains supplementary material, which is available to authorized users.

## Background and need for innovation

The 2019 novel coronavirus (COVID-19) pandemic disrupted traditional educational practices and created a decidedly abnormal learning environment for clinical medical students. Given that clinical rotations are in-person, bedside experiences, no model or infrastructure existed for their remote completion when they were suspended per the Association of American Medical Colleges’ (AAMC) recommendation on March 17, 2020 [1]. As a result, clinical-level medical students at the University of Michigan Medical School (UMMS) simultaneously experienced a sudden void in their education and a desire to understand the very pandemic that had upended their experiences.

Many medical education entities, at both the undergraduate and graduate levels, quickly adapted to fill this gap. These adaptations included the expansion of didactic curricula and surgical simulations for residents who had less operating room time [2, 3], conversion of in-person clerkship trainings and assessments to online [4, 5], and development of online repositories of COVID-19-related information for self-teaching [6, 7]. Although unique, none of these interventions were de novo curricula on pandemics tailored specifically to meet the educational needs of clinical medical students.

At first, clinical medical students at UMMS looked to the administration and faculty experts to provide a formal opportunity for continued learning during the pandemic. However, this traditional approach to curriculum development [8], wherein faculty experts are content suppliers and students are consumers [9], was not feasible at our institution during the early days of the pandemic, as the majority of our faculty were redeployed to assist with COVID-19-related efforts. Although Kern’s model remains the most well-known approach to curriculum development, there is a growing literature that describes the potential for learner involvement in this process. Student feedback is increasingly integrated during the development of medical school curricular content and structures [10–14]. Although evaluations of such processes have demonstrated that student involvement is valued by both students themselves [10, 11] and administrators [15], there is limited research describing the process and outcomes of a truly student-generated curriculum. It was in this context that a team of medical students developed a pandemics and medicine course that served as the only formal curriculum for clinical medical students at our institution during the pandemic.

## Goal of innovation

The first goal of this paper is to share with the medical education community the innovative approach that our students took to fill an urgent curricular need at our institution. Additionally, as this was a truly student-designed and -led effort under immense time constraints, there was no pre-emptive discussion about learning theory. Interestingly, however, as the students embraced their role as teachers, they began to inherently adopt the Master Adaptive Learner framework and other key tenets of adult learning theory to guide their curriculum development process. This paper describes how the disruption of traditional pedagogy resulted in an opportunity for a different scheme of student learning and partnership with faculty.

## Steps taken for development and implementation of innovation

### Medical students as curriculum creators

The COVID-19 pandemic revealed that a formal education in pandemic preparedness and response has not traditionally been emphasized in undergraduate medical education. Clinical-level students at UMMS, sent home without instructions, initially responded with attempts to self-teach about pandemics through news outlets, social media, and discussions with peers. Such conversations inspired one student (JBK) to draft an outline of a pandemic curriculum that used COVID-19 as a case study. As no such content existed publicly at the time, the proposed topics were based on information that she and classmates encountered and judged imperative to understand. To ensure that the topics addressed the knowledge gaps of the target audience, they were discussed during an open, school-wide needs assessment on continuing medical education during the pandemic. The resulting edits incorporated the opinions of the 84 student and faculty attendees of the meeting, and the final outline was approved by the administration. The student lead then recruited two additional student co-leads (CAK, NHM), a faculty advisor (EDL), and 13 medical student author volunteers to assist with curriculum development. Some of these student authors were identified through existing UMMS student interest groups, and others were selected according to self-reported relevant backgrounds and experiences.

### A by students, for students approach

The course was organized into five modules, and the student authors self-assigned to each module based on interest for the purpose of mastery. The resulting five groups of 2–4 authors curated content for their respective modules. Whenever possible, they cited the available evidence-based literature, and peer-reviewed articles were incorporated directly into the curriculum where relevant. However, as conventional, peer-reviewed COVID-19 publications were sparse in the early stages of the pandemic, student authors were also encouraged to navigate online news sources and social media for materials they found reliable, engaging, and important. This allowed them to simultaneously sidestep the dearth of formal scientific literature and rapidly access information from non-traditional sources. For example, Twitter (San Francisco, CA) is often the first place to find new information generally, and the pandemic was no exception [16]. The #MedTwitter community was particularly active during the early days of the pandemic and served as a space for trainees and physicians to connect, share information, and learn. Much of this content informed or was cited in the curriculum.

### Operationalizing the pandemics and medicine curriculum

The student-drafted materials were edited by the leadership team of three students (JBK, CAK, NHM) and one faculty member (EDL) for cohesiveness and then reviewed by 25 content experts. These experts included emergency medicine, critical care, and general medicine faculty; epidemiologists; physicians with palliative care training; medical educators with expertise on teaching concepts about evidence-based medicine; clinical ethicists; and health sciences librarians. They thoroughly reviewed the material through multiple iterations of edits to ensure that the content curated by the students was accurate, comprehensive, and well-supported. They proposed alternative sources where necessary and offered clinical insights where relevant, and they reviewed any non-traditional sources to confirm their veracity. The resulting curricular framework had short portions of student-written, expert-reviewed text that guided learners through relevant peer-reviewed articles, podcasts, webpages, charts, and videos. Learners were exposed to a variety of different types of mixed media and, where possible, were able to choose which type of content best fit their learning style.

The curriculum is composed of five modules (see Fig. 1 in Electronic Supplementary Material [ESM]): (1) pandemic/disaster response: systems-level principles, (2) pandemic/disaster response: at the bedside, (3) communication and mental health during a pandemic/disaster, (4) epidemiology, and (5) finding and verifying evidence-based information during a pandemic. To augment learning, the student authors proposed and designed knowledge and thought exercises for their peers. The first four modules each include 4–6 multiple-choice questions (MCQs) for formative self-assessment, and learners were also required to publish an original post to an asynchronous discussion board in response to any of four discussion prompt options per module. To encourage peer-to-peer learning, learners then responded to at least one peer’s post. The MCQs and discussion board responses were graded for completion. Modules 1 and 5 include additional deliverables consisting of (1) a short essay regarding health inequities in pandemics and (2) a one-page, cited infographic about a COVID-19 topic of the learner’s choice, respectively. Learners received individualized feedback from faculty on these two deliverables, and faculty were provided with rubrics to assist in the grading process (see ESM, Fig. 2).

With assistance from the Office of Medical Student Education (OMSE), the material was transferred to the UMMS online educational platform Canvas by Instructure (Salt Lake City, UT). This platform was chosen because it supports embedded articles and videos, has built-in multiple choice assessment capabilities, and allows for the incorporation of discussion boards into the curriculum. The resulting online, 40-hour curriculum was launched on April 13, 2020, just 17 days after the original outline was proposed. It was required for and completed by all 371 active second- and third-year clinical students at UMMS. Fourth-year students had access to the curriculum but were not mandated to complete it because of their impending graduation. To support the diversity of student needs and well-being at the beginning of the pandemic, students were given the flexibility to complete the course on their own schedule during an 8‑week period.

### Student authors as master adaptive learners

It is important to note that our approach, while unorthodox, is well-founded in adult learning theory. The Master Adaptive Learner (MAL) framework, a conceptual model whereby adult learners continuously identify, react to, and reassess their learning against a perceived knowledge gap, is characterized by four phases: *planning, learning, assessing*, and *adjusting* [17]. The development of this course serves as a case study in how our student authors innately engaged in the MAL framework, even without prior intention of doing so. They engaged in the *planning* phase as they reflected on pre-existing knowledge about their respective modules and pandemics in general, identified their knowledge gaps, and reasoned that what they had learned thus far was insufficient in the new paradigm created by COVID-19. They then approached the *learning* phase by actively seeking out new information and critically challenging current assumptions about how medicine is practiced given these extenuating circumstances. In doing so, the student leads and authors served as self-regulated, successful adult learners, both for their own benefit as well as for that of their classmates. As not all of our learners have served COVID-19 patients yet in the clinical space, the *assessing* and *adjusting* phases are still forthcoming.

### Student authors as self-directed learners

The work of the student authors and their approach to the development of this curriculum also fits neatly into the theory of self-directed learning. The theory posits that “individuals take the initiative, without the help of others” to diagnose their learning needs, formulate learning goals, and drive the selection of content, delivery methods, and assessment of their learning [18]. A notable strength of self-directed learning is that it facilitates purposeful learner engagement in situations of isolated activities [19]. During an active pandemic, which relegates education to sequestered environments, the intentional incorporation of self-directed learning may be more timely than ever. Furthermore, following the resolution of the pandemic, traditional education delivery may retain more virtual components than before.

## Outcomes of innovation

### Student learners’ perception is positive

We designed a post-survey via Qualtrics (Provo, UT) to gauge learners’ perceived gains in knowledge, preparedness, comfort, and understanding regarding pandemics after completing the curriculum (see ESM, Fig. 3). The survey consisted of 12 five-point Likert-scale questions and 6 demographics questions. At the request of our administration and due to concern about survey burnout during the height of the pandemic, students could opt in or out of use of their anonymously collected responses for scholarship. The entire study (HUM00180122), including this survey and subsequent analyses, was determined to be exempt by the University of Michigan Institutional Review Board on April 7, 2020. Microsoft Excel (Redmond, WA) was used to tabulate responses.

Two hundred and sixty-five (71%) of 371 learners permitted use of their survey responses in analysis. When responses to the ten Likert questions that assessed knowledge, understanding, comfort, and preparedness were averaged, a majority (68%) of learners agreed (defined as a response of either “agree” or “highly agree”) that their sentiments improved upon completion of the course. When individual questions were considered, learner understanding increased the most with regard to pandemic terminology, resource allocation in the face of scarcity, and the roles of various organizations in addressing pandemics (79%, 78%, and 77% agreement, respectively) (see ESM, Table 1). In contrast, the course was least effective at increasing learners’ sense of preparedness to practice as a resident during a future pandemic (52% agreement). Notably, almost all learners (93%) felt that this content is relevant to all medical students, and 90% felt that the relevance extends across all specialties.

## Critical reflection

### Limitations

We used Kirkpatrick’s evaluation model to assess the reaction of the learners (i.e., Kirkpatrick’s first level) [20]. Although we recognize that this level of evidence limits our readers’ ability to extrapolate the strength of this curriculum, this was a choice we intentionally made: with 17 days to develop both a curriculum and a methodology for evaluating that curriculum, our team chose to dedicate the majority of its effort to providing our students with a strong product to fill an educational gap. It was simply not possible to simultaneously produce a robust, higher-level evaluation tool given those constraints. We were also acutely aware of student well-being during the early stages of the pandemic; students were already overwhelmed by abrupt disruptions to their lives, and we felt that administering additional or longer evaluation tools would further detract from their well-being. This concern for survey burnout is also why, at the request of the medical school administration, no open-ended questions were asked. Finally, it is important to acknowledge that 29% of learners did not opt to include their survey responses in data analysis, and the resulting sample may be biased.

Additionally, this approach to curriculum development arose during a period of educational upheaval, and there may be limitations to its applicability during “normal” times. Because learning and many other engagements were paused at the outset of the pandemic, student authors were able to devote themselves rather singularly to this project. Finally, the success of this project was contingent on the UMMS administration’s acceptance of the unconventional approach.

### Concluding thoughts

With these limitations in mind, we feel that the most important contribution this piece makes to the literature is one of process: students can contribute to the development of their own curricula during extenuating circumstances, and a Kirkpatrick’s first-level evaluation of the resulting curriculum suggests that target learners view it favorably. In particular, the fact that almost all learners found the course relevant to all medical students suggests that the curriculum fills a need at the undergraduate medical education level. The relative lack of perceived relevance to residency is also to be expected, as the content was specifically catered towards the average undergraduate medical student knowledge level.

The successful implementation of this student-driven model for curriculum development illustrates that although the traditional, faculty-designed approach may be appropriate in some settings, exclusive reliance on that approach may in fact limit student learning opportunities. By incorporating students into the curriculum development process, their learning is not restricted to the receipt of a finalized curriculum alone; rather, they are able to learn while actively developing the product. Faculty remain integral to the process, but in a role that positively disrupts the traditional teacher-learner hierarchy. Rather than serving as the exclusive providers of information, faculty in this model facilitate a framework that drives students to pursue the information themselves, allowing for synergistic learning opportunities between both parties. Because the resulting product is created by students, their peers will also benefit from a curriculum that is likely more student-focused than one derived from a faculty-only team.

In light of these potential benefits to both the student developers and their peer learners, other institutions that hope to adopt this approach should be encouraged to give full autonomy to students from the project’s outset. We also recommend that peer institutions consider the use of non-traditional sources (such as social media and preprint articles) when appropriate. Not only does the incorporation of such sources allow for the inclusion of more mixed media content into the resulting course, but it also empowers students to critically evaluate the legitimacy of such sources. This is an important learning outcome in and of itself, and it also presents an additional opportunity for partnership between students and faculty as they assess the veracity of the content.

One key potential pitfall to consider is whether there is a long-term goal of publishing the curriculum to make it available to other institutions. When we began to pursue publication, we were hindered by a myriad of copyright laws that applied once we attempted to share the content with those not covered by our own institution’s copyright privileges. Finally, if other institutions have more time to develop their student-generated curricula, we would encourage the development of a more robust evaluation tool.

While our results were limited to Kirkpatrick’s first level of evaluation, the positive reactions of those who took the course suggest an interest in future related learning [20]. We are working to deploy an online, open-access version of the content, and we will use the MAL framework to guide revisions to the pandemic curriculum. Moving forward, as our student authors and learners return to the clinical arena, their reflections will inform the curriculum updates, thereby completing the *assessing* and *adjusting* phases of the MAL cycle. We intend to employ more high-level evaluation tools (e.g., pre/post assessment of knowledge and skills, interviews and/or observations of students as they apply new knowledge to clinical practice, or eliciting patient feedback on student behaviors) at our own institution as we launch future planned iterations of this curriculum.

## Supplementary Information

Acknowledgements

**Fig. 1. **Pandemics and medicine curriculum: Course outline

**Fig. 2. **Faculty grading rubrics for assignments in Modules 1 and 5

**Fig. 3**. Post-course survey questions

**Table 1. **Learner (N = 265) responses to post-course Likert survey
