# Supplementary material for: Coping with COVID-19: medical students as strong and responsible stewards of their education
Source: Perspect Med Educ. 2021 Jan 25;10(3):187–91. doi: 10.1007/s40037-021-00650-3 (PMC7829638; doi:10.1007/s40037-021-00650-3)
Supplement: Supplementary file 1 — Acknowledgements [file 40037_2021_650_MOESM1_ESM.docx]

**Acknowledgements**

The authors would like to express gratitude to our Student Authors: Zoey Chopra, Erin Finn, Rebecca Howland, Benjamin Li, Isabel Lott, Annie Murphey, Noah Newman, Stephanie Reyes, Dr. Jessica Santos-Parker, Dr. Keli Santos-Parker, Eric Steinbrook, Nithya P. Vijayakumar, Ann Wolski; and our Faculty Content Experts: Drs. Mahshid Abir, Andrew Barnosky, Ben Bassin, Khalil Chedid, Cynthia Chen, Ivan Co, Gregory Dalack, Peter England, Julieann Grant, Carrie Harvey, Jack Iwashyna, Cornelius James, Kathleen Li, Marcia Perry, Nana Sefa, Graham Smith, Laura Taylor, and Ryan Tucker, as well as Emily Ginier, John Kubale, Gurpreet Rana, Jean Song, and Whitney Townsend. In addition, the authors would like to acknowledge the efforts of Dr. Patrick Bridge, Samiah Haque, and Dr. Meg Wolff, and special appreciation to Dean Emeritus James Woolliscroft and Dr. Larry Gruppen. Finally, the authors thank the University of Michigan Medical School leadership: Deans Michelle Daniel, Rajesh Mangrulkar, and Seetha Monrad; and OMSE staff Tomas Mauricio and Heather Wagenschutz.
