# Supplementary material for: Coping with COVID-19: medical students as strong and responsible stewards of their education
Source: Perspect Med Educ. 2021 Jan 25;10(3):187–91. doi: 10.1007/s40037-021-00650-3 (PMC7829638; doi:10.1007/s40037-021-00650-3)
Supplement: Supplementary file 2 — Fig. 1. Pandemics and medicine curriculum: Course outline [file 40037_2021_650_MOESM2_ESM.docx]

**Figure 1. Pandemics and medicine curriculum: Course Outline**

Timeline of events since the start of the pandemic

Evidence-based medicine overview

Levels of evidence

Outbreak terminology

What is epidemiology?

The concepts behind the curves

Palliative care

Talking to patients about COVID-19

Mental health of patients and special populations during a pandemic

Mental Health of Health Care Providers during a pandemic

PPE and preventing transmission of pathogens

Triage principles

Approach to the dyspneic patient with concern for COVID-19

Lung point of care ultrasound (POCUS) in pulmonary COVID-19

Ethics and resource allocation in the context of a pandemic

Disaster response at a systems level

Current responses to COVID-19 pandemic: Case studies from other countries

Lessons learned from past pandemics

Finding Healthcare Equity in Pandemic Conditions

**Module 5**

**Finding and Verifying Evidence-Based Information During a Pandemic**

**Module 4**

**Epidemiology**

**Communication and Mental Health During a Pandemic/Disaster**

**Module 3**

**Module 2**

**Pandemic/Disaster Response: At the Bedside**

**Module 1**

**Pandemic/Disaster Response: Systems-Level Principles**
