# Supplementary material for: Coping with COVID-19: medical students as strong and responsible stewards of their education
Source: Perspect Med Educ. 2021 Jan 25;10(3):187–91. doi: 10.1007/s40037-021-00650-3 (PMC7829638; doi:10.1007/s40037-021-00650-3)
Supplement: Supplementary file 3 — Fig. 2. Faculty grading rubrics for assignments in Modules 1 and 5 [file 40037_2021_650_MOESM3_ESM.docx]

**Figure 2: Faculty grading rubrics for assignments in Modules 1 and 5**

| **Evaluation Rubric for Faculty Reviewers: End of Module 1 Assignment: Health Equity**  The student…  [Yes (2 pts), No (0 pts), Unclear (1pt) for each element]   1. Listed which social determinant of health (SDoH) condition they selected 2. Described how the pandemic is impacting/exacerbating the selected SDoH condition 3. Described strategies to address/mitigate the selected SDoH at local and national level 4. Gave at least one (1) zip code in Washtenaw County that carried a disproportionate number of COVID-19 cases: https://www.washtenaw.org/3108/Cases 5. Explained at least one (1) systemic force they think contributed to this finding.   Faculty formative feedback comments: __________________________________________________  Total Points: ____ (0-10)  > / = 5 points is Satisfactory; < 5 points - discuss what is missing with the student (and have them revise and resubmit the assignment) |
| --- |

| **Evaluation Rubric for Faculty Reviewers: End of Module 5 Assignment: Infographic**  The student…  [Yes (2 pts), No (0 pts), Unclear (1pt) for each element]   1. Created a clear, well-organized 1-page infographic summary of the question and their analysis of the evidence 2. Performed an adequate literature and source search (Methods document; 5 citations)    1. Indicated where sources were found and the target audience (1 pts)    2. Identified 5 citations (1 pts)    3. Describe each study using 5S pyramid (1 pts)    4. Identifies study limitations and relationship to COVID-19 pandemic (1 pts) 3. Evaluated and Analyzed the literature and sources selected in a rigorous manner    1. Explains whether the data supports the conclusions in each source 4. Discussed limitations identified in the sources cited as well as those excluded from analysis     Faculty formative feedback comments: __________________________________________________  Total Points: ____ (0-10)  > / = 5 points is Satisfactory; < 5 points - discuss what is missing with the student (and have them revise and resubmit the assignment) |
| --- |
