# Supplementary material for: Coping with COVID-19: medical students as strong and responsible stewards of their education
Source: Perspect Med Educ. 2021 Jan 25;10(3):187–91. doi: 10.1007/s40037-021-00650-3 (PMC7829638; doi:10.1007/s40037-021-00650-3)
Supplement: Supplementary file 4 — Fig. 3. Post-course survey questions [file 40037_2021_650_MOESM4_ESM.docx]

**Figure 3. Post-Course Survey Questions**

| This survey uses a scale of 1 (highly disagree) to 5 (highly agree). Please answer as honestly as possible.   1. This curriculum significantly increased my **knowledge** regarding the terminology used to discuss pandemics. 2. This curriculum significantly increased my **knowledge** regarding the proper use of personal protective equipment. 3. This curriculum significantly increased my **understanding** of the roles of various local, state, and federal organizations in addressing pandemics. 4. This curriculum significantly increased my **understanding** of resource allocation in periods of space and equipment scarcity. 5. This curriculum significantly increased my **understanding** of health equity challenges in the setting of a pandemic. 6. This curriculum significantly increased my **comfort** with educating my patients, family, and friends aboutCOVID-19 and pandemics in general. 7. This curriculum significantly increased my **comfort** with participating in healthcare team discussions regarding the care of a COVID-19-affected patient. 8. This curriculum significantly increased my **comfort** with participating in goals of care discussions with COVID-19-affected patients and their families. 9. This curriculum significantly increased my **comfort** with navigating a rapidly changing and complex medical research literature. 10. This curriculum significantly increased my **preparedness** to practice as a resident during a future pandemic. 11. The content of this curriculum is **relevant** to all physician specialties. 12. The content of this curriculum is **relevant** to all medical students. 13. How old are you? Please enter two digits only, no text. [free text box] 14. Regarding gender, please choose the option with which you most closely identify.   [ ] male [ ] female [ ] non-binary [ ] other, please indicate: [free text box]   1. What year in medical school training are you? Please check one.   [ ] M2   [ ] M3  [ ] M4 [ ] Other, please indicate: [free text box]   1. What is your intended specialty at this time? [drop-down selection]   Anesthesiology  Dermatology  Emergency Medicine  Family Medicine  Internal Medicine  Neurology  Obstetrics & Gynecology  Ophthalmology  Orthopedic Surgery  Otolaryngology  Pathology  Pediatrics  Physical Medicine & Rehabilitation  Plastic Surgery  Psychiatry  Radiology  Surgery - General  Urology  Undecided   1. Which of the following rotations and/or sub-internships have you completed? Please check all that apply. If you were on one of these rotations but were removed due to the COVID-19 pandemic, please still check the box.   [ ] Internal Medicine  [ ] Surgery  [ ] Pediatrics  [ ] Ob/Gyn  [ ] Neurology  [ ] Family Medicine  [ ] Psychiatry  [ ] Emergency Medicine  [ ] Intensive Care Unit (ICU)  [ ] Emergency Critical Care Center (EC3)   1. Consider your experiences, education, and/or advanced degree(s) **outside of your medical school education.** Do you feel any of these helped you navigate the pandemic curriculum modules? Please check all that apply.   [ ] Epidemiology  [ ] EMT  [ ] Clinical Research  [ ] Military  [ ] Public Health  [ ] Other, please indicate [free text box]  [ ] **Yes, I consent** to the use of my responses in this survey for the improvement of this curriculum and possible future published works.  [ ] **No, I do not consent** to the use of my responses in this survey for the improvement of this curriculum and possible future published works. |
| --- |
