# Supplementary material for: Coping with COVID-19: medical students as strong and responsible stewards of their education
Source: Perspect Med Educ. 2021 Jan 25;10(3):187–91. doi: 10.1007/s40037-021-00650-3 (PMC7829638; doi:10.1007/s40037-021-00650-3)
Supplement: Supplementary file 5 — Table 1. Learner (N = 265) responses to post-course Likert survey [file 40037_2021_650_MOESM5_ESM.docx]

**Table 1. Learner (N=265) responses to post-course Likert survey**

| **Survey Question** | **Agree or Highly Agree: N (%)** | **Neutral: N (%)** | **Disagree or Highly Disagree: N (%)** |
| --- | --- | --- | --- |
| **The content of this curriculum is relevant to:** | | | |
| All medical students. | 245 (92.5%) | 10 (3.8%) | 10 (3.8%) |
| All physician specialties. | 238 (89.8%) | 20 (7.5%) | 7 (2.6%) |
| **This curriculum significantly increased my:** | | | |
| Knowledge regarding the terminology used to discuss pandemics. | 208 (78.5%) | 37 (14%) | 20 (7.5%) |
| Understanding of resource allocation in periods of space and equipment scarcity. | 206 (77.7%) | 39 (14.7%) | 20 (7.5%) |
| Understanding of the roles of various local, state, and federal organizations in addressing pandemics. | 204 (77%) | 49 (18.5%) | 12 (4.5%) |
| Understanding of health equity challenges in the setting of a pandemic. | 199 (75.1%) | 43 (16.2%) | 23 (8.7%) |
| Comfort with educating my patients, family, and friends about COVID-19 and pandemics in general. | 194 (73.2%) | 45 (17%) | 26 (9.8%) |
| Comfort with participating in healthcare team discussions regarding the care of a COVID-19-affected patient. | 181 (68.3%) | 55 (20.8%) | 29 (10.9%) |
| Comfort with participating in goals of care discussions with COVID-19-affected patients and their families. | 173 (65.3%) | 66 (24.9%) | 26 (9.8%) |
| Comfort with navigating a rapidly changing and complex medical research literature. | 162 (61.1%) | 64 (24.2%) | 39 (14.7%) |
| Knowledge regarding the proper use of personal protective equipment. | 143 (54%) | 73 (27.5%) | 49 (18.5%) |
| Preparedness to practice as a resident during a future pandemic. | 138 (52.1%) | 66 (24.9%) | 61 (23%) |
